# Green Method for the Selective Electromembrane Extraction of Parabens and Fluoroquinolones in the Presence of NSAIDs by Using Biopolymeric Chitosan Films

**DOI:** 10.3390/membranes13030326

**Published:** 2023-03-12

**Authors:** Cristina Román-Hidalgo, María Jesús Martín-Valero, Germán López-Pérez, Mercedes Villar-Navarro

**Affiliations:** 1 Department of Analytical Chemistry, Faculty of Chemistry, Universidad de Sevilla, c/Prof. García González, s/n. 41012 Seville, Spain; 2 Department of Physical Chemistry, Faculty of Chemistry, Universidad de Sevilla, c/Prof. García González, s/n. 41012 Seville, Spain

**Keywords:** parabens, fluoroquinolones, NSAIDs, chitosan, electromembrane extraction

## Abstract

A chitosan biopolymeric membrane was successfully used as a support in a green electromembrane extraction procedure for the simultaneous and selective extraction of seven parabens and three fluoroquinolones in the presence of three non-steroidal anti-inflammatory drugs. The optimal experimental conditions (10 mL donor phase and 50 μL acceptor phase, pH 10 in both phases; 80 V of applied voltage during 15 min of extraction time) were determined, providing high enrichment factors for six of the studied parabens (EF ≥ 90) and the three fluoroquinolones (EF ≥ 50). Wide linear concentration ranges (0.5–500 μg L^−1^), good linearity (>97%), low limits of detection (0.2–1.1 μg L^−1^), and good repeatability (relative standard deviation values 4–10%) were achieved. The proposed method was successfully applied for the extraction of the target analytes from different kinds of water samples (river, lake, and swimming pool). The usage of a chitosan membrane in the extraction process presents many advantages, as it is a biodegradable and versatile support, offering a good alternative to commercial plastic materials commonly used in this methodology and these procedures.

## 1. Introduction

In recent years, pharmaceuticals and personal care products (PCPs) have come to be considered as contaminants of emerging concern (CECs). Different pharmacologically active drugs, such as non-steroidal anti-inflammatory drugs (NSAIDs), as well as fluoroquinolones (FQs) used as broad-spectrum antibiotics, are frequently detected in surface water, wastewater, or even treated water for human consumption [1,2].

Following the COVID-19 pandemic period, there has been an important increment in the consumption of pharmaceuticals, which has led into an increasing presence of these kinds of compounds in untreated sewage and in effluents from waste water treatment plants (WWTP) [3]. Meanwhile, parabens (*p*-hydroxybenzoates) have been widely used as preservatives in PCPs, cosmetics, and drugs, due to their antibacterial properties. Although their usage in cosmetics has been regulated by the European Union (EU) because these compounds are considered to be weak endocrine disruptors [4], elevated levels of PCPs have also been found in water treated for consumption. Consequently, the scientific community is becoming increasingly concerned about the impact of these kinds of chemicals on the environment. In fact, such materials have gone from being called “micro-pollutants” to being renamed as CECs. In this context, optimization of analytical methods which enable the selective quantitation of these compounds has emerged as a challenge in the field of the analytical chemistry.

Liquid phase microextraction (LPME) techniques, in any of their multiple configurations, represent a valuable tool for the extraction and determination of pharmaceuticals and parabens [5,6,7]. In particular, electromembrane extraction (EME) systems have been successfully used for this purpose. In EME, the extraction of target analytes is achieved by electrokinetic migration under the action of an external electric field. In the initial step, compounds of interest (in ionized form) migrate from sample (donor phase) towards an organic phase (5–25 μL), which constitutes the supported liquid membrane (SLM). The SLM impregnates a porous support (usually a polymeric flat membrane or fiber). In a second step, the analytes migrate towards an acceptor solution, where the oppositely charged electrode is located. By controlling the magnitude and direction of the applied voltage, high selectivity can be achieved using EME methods [8]. Several additional advantages such as low solvent consumption and capability for using new materials for supports as alternatives to plastics have also been remarkable [9,10,11].

Indeed, several strategies have been implemented to improve extraction efficiency in EME, mainly related to the SLM itself or to the type of support employed. Gels of different chemical natures, ionic carriers, and modern solvents such as deep eutectic solvents or ionic liquids have been employed [12,13]. Additionally, novel materials have appeared for use as supports, offering favourable alternatives to those conventionally prepared with a plastic base (mainly polypropylene (PP)), including nanoparticles, polymer inclusion membranes, nanostructured composites, or biopolymers [14].

Within this field of industry, several biopolymers have been employed in EME systems. Notably, agarose (both film and gel), polyacrylamide, or tragacanth gel membranes have been described as excellent green supports for different applications in these extraction procedures [15,16,17,18].

On the other hand, chitosan has emerged as a promising natural, versatile, and biodegradable material for use in EME systems. Membranes constructed of this polymer enable the physical separation of donor and acceptor phases without the need for any organic solvent as liquid membrane, which constitutes a very important advantage in the context of green chemistry. Moreover, due to the physical and chemical features of chitosan, films can be synthesized showing excellent mechanical properties (flexibility, permeability, hardness, ease of handling). Furthermore, the chemical structure of this biopolymer offers different linkage points through amine and hydroxyl groups, providing a very useful tool to address the extraction of basic and acidic compounds. This feature makes chitosan a very versatile raw material that allows the synthesis of tailor-made membranes aimed at the selective extraction of targeted analytes. Indeed, chitosan films have already been successfully used in different EME procedures for the extraction and determination of NSAIDs and polar acidic drugs [19], as well as for the selective extraction of FQs from urine samples [10]. Regarding paraben compounds, the versatile chemical structure of chitosan has been exploited for the fabrication of a novel molecularly imprinted biomembrane, capable of cleaning and isolating paraben families from complex matrices by means of advanced matrix solid-phase dispersion [20]. Nevertheless, there is no evidence in the revised literature of any EME procedure where chitosan has been used for the selective extraction of parabens. Conversely, the extraction of NSAIDs, FQs, and parabens has been addressed separately [14]. Simultaneous or selective EME of compounds belonging to these drug families remains as a challenge for contemporary analytical methods.

For this reason, a biopolymeric chitosan membrane was used in this work as an environmentally friendly support for the selective extraction of parabens and FQs in the presence of NSAIDs, as a greener approach to EME avoiding the traditional usage of an organic solvent. A set of seven different parabens compounds: methyl 4-hydroxybenzoate (MeP), ethyl 4-hydroxybenzoate (EtP), propyl 4-hydroxybenzoate (PrP), isopropyl 4-hydroxybenzoate (iPrP), butyl 4-hydroxybenzoate (BuP), isobutyl 4-hydroxybenzoate (iBuP), and benzyl 4-hydroxybenzoate (BzP), and three FQs: marbofloxacin (MRB), enrofloxacin (ENR), and flumequine (FLM) were selectively extracted in the presence of three NSAIDs: ketoprofen (KTP), naproxen (NAX), and salicylic acid (SAL). The influence of the main experimental parameters governing the extraction procedure has been studied using a Box–Behnken experimental design. Enrichment factors (EFs) for each extracted analyte were used as the response factor to achieve the optimal experimental conditions for performing the procedure. The application of the proposed methodology for the determination of parabens and FQs in the presence of NSAIDs was successfully achieved with different kinds of water samples (river, lake, and swimming pool), providing EFs that were higher than those reported in the literature. The proposed method can be considered a green analytical procedure because organic solvent is not involved. Furthermore, the membrane is synthetized from chitosan, a biodegradable polymer, providing a suitable alternative to plastic traditional supports in EME procedures.

## 2. Materials and Methods

### 2.1. Chemicals and Reagents

Chemicals and reagents used in this research were of analytical grade. Chitosan of 310,000–375,000 Da molecular weight, MeP, EtP, PrP, iPrP, BuP, iBuP, BzP, KTP, NAX, SAL, MRB, ENR, and FLM were acquired from Fluka-Sigma-Aldrich (Madrid, Spain). HPLC-grade methanol, acetic acid, and hydrochloric acid were obtained from Merck (Darmstadt, Germany). Sodium hydroxide and sodium dihydrogen phosphate were obtained from Panreac (Barcelona, Spain). Working standard solutions of parabens, NSAIDs, and FQs were daily prepared by adequate dilutions in ultrapure water from methanolic stock solutions (400 mg L^−1^). A Milli-Q water purification system (Millipore, Bedford, MA, USA) was employed to provide ultra-high-purity water.

### 2.2. EME Procedure

The synthesis of biopolymeric chitosan membranes for use in EME techniques was optimized in previous research [10,21]. Therefore, the biofilm was prepared with 60% (*w*/*w*) of chitosan and 40% (*w*/*w*) of Aliquat^®^336. Pieces of the obtained films (5 × 5 mm) were glued to the end of a glass tube (2 mm internal diameter and 2 cm length). The acceptor phase (50 μL), adjusted to pH 10 by adding an aqueous NaOH solution, was placed inside the prepared capillary tube. A volume of 10 mL of donor phase at pH 10, containing an aqueous solution of parabens FQs and NSAIDs (at a concentration of 100 μgL^−1^) was placed in a vial. After preparing the acceptor and donor phases, the EME device was assembled as follows: the tube containing the acceptor phase was placed inside the vial containing the donor solution. Two platinum electrodes (0.25 mm diameter each with a separation of 2 mm between them) were introduced in the acceptor and donor solutions, respectively. A three-channel laboratory DC power supply, with programmable voltage in the range 1–120 V (Benchtop Instrument, Pennsylvania, USA) was used for connecting both electrodes. To monitor the electric power consumption during the EME process, a digital multimeter (3430 4 ½-digit PeakTech^®^, Ahrensburg, Germany) was employed. Data acquisition during the extraction period was monitored using an automatized system controlled by a personal computer. The best extraction efficiency was obtained applying a DC potential of 80 V for 15 min with constant stirring at 600 rpm. The self-made device for carrying out the EME procedure is shown in Figure S1.

### 2.3. HPLC-DAD Chromatographic Determination

Parabens, FQs, and NSAIDs were separated and determined using an Agilent (Palo Alto, CA, USA) 1100 series liquid chromatograph equipped with a diode array detector (DAD), a fluorescence detector (FL), an injector with a 20 µL-loop, a quaternary pump, a vacuum degasser, and a thermostatic column compartment. A Kromasil^®^ 100 Å, C18, 3.5 µm (15 mm × 4.6 mm i.d.) (Schrarlab S.L., Barcelona, Spain) was used as a guard column. Elution and separation of the target analytes were carried out on an Eclipse^®^ XDB-C18 3.5 µm (150 mm × 3.0 mm i.d) (Agilent Technologies, Little Falls, DE, USA) chromatographic column, thermostatically controlled to 25 °C.

Gradient elution was optimized using mobile phase A (28 mM aqueous phosphate buffer pH 2.5) and mobile phase B (methanol) for the separation of the target analytes, requiring 0.4 mL min^−1^ flow rate. Table S1 (in supplementary material) indicates the optimized gradient elution. For quantitation purposes, the following monitoring wavelengths for each compound were used: 255 nm for all parabens, FLM, and KTP, 300 nm for MRB, 280 nm for ENR, 235 nm for SAL, and 230 nm for NAX. 

### 2.4. Box–Behnken Experimental Design for EME 

The experimental factors governing EME procedure were optimized using a Box–Behnken design (MODDE statistical software, version 13.1 (MKS Umetrics AB, Sweden)), in order to reach the maximum EF of the target analytes. Taking into account previous results, the most important factors affecting the EME procedure were donor- and acceptor-phase pH, applied voltage, and extraction time, thus these were selected as key factors, with EF the variable set for analytical response.

### 2.5. Greenness Assessment

Green analytical chemistry (GAC) metrics were applied in order to assess the greenness profile of the proposed EME method, and other sample preparation methods previously reported in the literature, for comparative purposes. The principles of GAC were evaluated and individually scored for each of the studied methods. Therefore, a final overall score was assigned, representative of the method’s suitability from an ecological point of view. Two greenness assessment tools, the Green Analytical Procedure Index (GAPI) [22] and the Analytical Greenness Metric for Sample Preparation (AGREEprep) [23], were applied.

### 2.6. Water Samples

Three water samples from different locations (lake, river, and swimming pool) were collected in summer. The lake water sample was collected from “Lagos del Serrano”, located in the foothills of the Sierra Morena, Guillena, Sevilla. Its main arteries are the Cala River and the reservoir of the same name. The river water sample was collected from a recreational area that takes advantage of the course of the Arroyomolinos river to form an artificial beach, located in Zahara de la Sierra, Cádiz. The sample of swimming pool water was collected from a social and sports center in the metropolitan area of Sevilla.

## 3. Results and Discussion

### 3.1. Chromatographic Conditions

**Figure 1:**
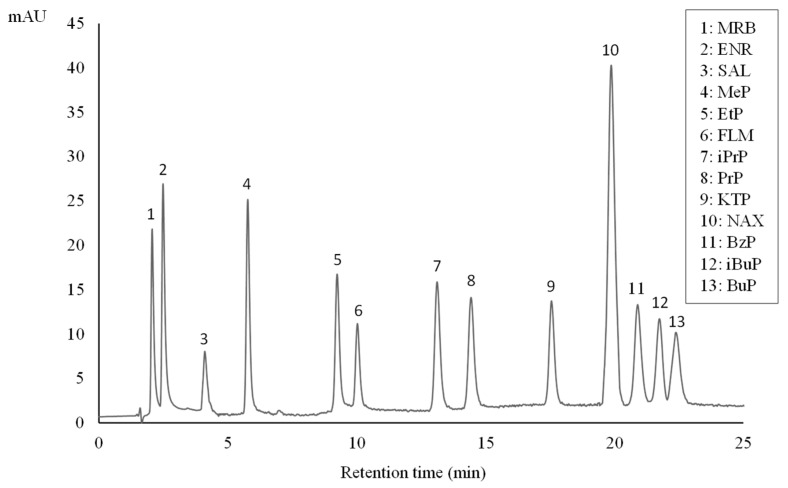
HPLC chromatogram of an aqueous standard solution (100 µgL^−1^) of parabens, FQs, and NSAIDs.

In accordance with the literature [24,25], a highly packed chromatographic column (Eclipse^®^ XDB-C18 3.5 µm) was selected for the HPLC separation and determination of the analytes of interest. In a first attempt, preliminary tests were performed using different mobile phases under isocratic conditions. Mixtures of acetonitrile/phosphate buffer (28 mM, pH 2.5) aqueous solution and methanol/28 mM phosphate buffer (pH 2.5) aqueous solution were tested in different ratios. Gradient elution was finally required for higher resolution separations as well as to ensure good peak symmetry. After optimization, the program of gradient elution described in Section 2.3 was the best choice to achieve good compromise in terms of peak shape, reproducibility, and analysis time. Figure 1 shows a chromatogram corresponding to a standard solution containing all the target analytes.

### 3.2. Optimization of EME Experimental Conditions

#### 3.2.1. Preliminary Assays

As has been discussed previously, the most important experimental parameters controlling EME procedures are pH, the volumes of the donor and acceptor phases, applied voltage, stirring speed, as well as extraction time. In previous work using this same kind of EME device [10,19,21], optimum volumes for both phases were settled, being 10 mL for donor solution and 50 µL for acceptor solution, as determined by the shape and size of the EME device. In addition, 600 rpm was fixed as the maximum stirring speed, to avoid vortices in the donor solution.

During this screening step, multiple experiments were carried out to select the appropriate ranges of the other experimental parameters mentioned above. These preliminary assays were focused on finding the best conditions for parabens extraction, as these compounds have not been previously studied with this EME configuration.

Firstly, it was observed that alkaline media in both donor and acceptor phases was necessary for the extraction to take place. This fact is in good agreement with the pKa values of the investigated parabens, indicating they are found in their ionic form in alkaline media (see Table S2), favoring migration through the chitosan membrane (positively charged by the presence of amine groups in the biopolymer structure) into the acceptor solution. In experiments carried out at pH < 8 in acceptor phase, no extraction of parabens was obtained, while pH < 8 values in donor phase led to very poor extraction. On the other hand, it is remarkable that paraben degradation products appeared above pH 12. In these experiments, the applied voltage was in the range 70–120 V and the extraction time varied between 5 and 40 min. 

It could be expected that an increase in applied voltage would lead to an improvement in extraction efficiency, as it enhances the migration of the analytes from donor to acceptor phase across the membrane. However, applied voltages higher than 100 V led to degradation of parabens. In addition, poor reproducibility due to instability of the EME system was observed. Furthermore, the extraction time in EME procedure is a key factor as it favors the transference process between the donor and acceptor phases. Accordingly, it was observed that times longer than 20 min did not lead to an improvement in EFs. Above 30 min, electrolytic phenomena occurred associated with a subsequent decrease in the extraction efficiency [26].

#### 3.2.2. Box–Behnken Design

If a Box–Behnken design is applied, an analytical response can be correlated with a number of independent factors by using a second order model. The number of experiments required to perform the analysis is calculated according to the following equation:

$$N=2\;K\;\left(K-1\right)+C$$

where K is the number of factors and C is the number of the experiments at the central point [27]. According to the obtained results from the preliminary assays, in the presented work there are four independent factors (K) for the model: the donor phase pH (8–12), the acceptor phase pH (8–12), the applied voltage (70–100 V), and the extraction time (5–20 min). By using C = 3, the Box–Behnken design requires N = 27 experiments. In this case, the analytical response investigated corresponds to the EF for each worked paraben. Experiments were carried out in randomized order to minimize possible effects of uncontrolled factors. Table 1 shows the complete design matrix, including the three experiments at the central point (marked as experiments number 25, 26, and 27 in the table) with the following coordinates: t = 12.5 min, V = 85 V, and pH = 10 for the donor and acceptor solutions, respectively.

**Figure 2:**
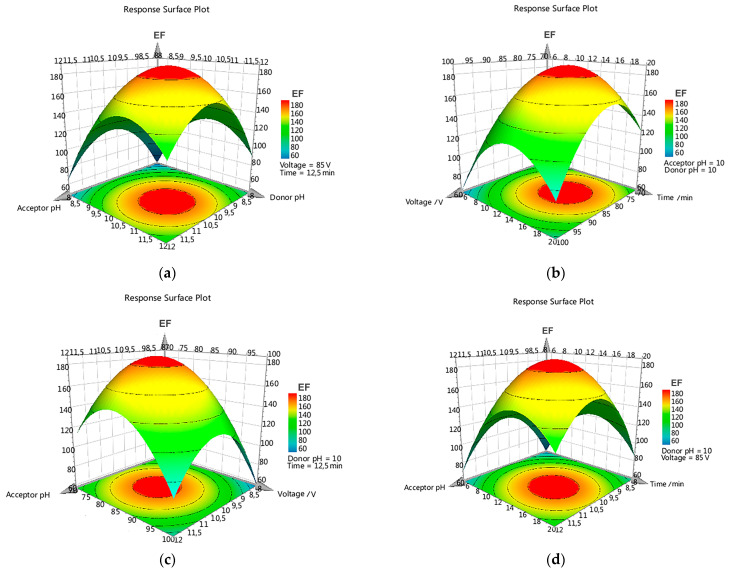
Three-dimensional response surface profiles for PrP. Plots of Enrichment Factor (EF, z-axis) as function of (**a**) acceptor phase pH and donor phase pH; (**b**) applied voltage and extraction time; (**c**) acceptor phase pH and applied voltage; (**d**) acceptor phase pH and extraction time. All other factors were set to their central value.

Response surface methodology (RSM) was conducted for all parabens, and the obtained three-dimensional plots were analyzed. In all cases, a very similar response was obtained for each paraben due to the resemblances in their chemical structures and observed properties, such as pKa values (8.17–8.40) or dipolar moments (0.614–0.930). Figure 2a–d displays the tridimensional response plots for PrP, as a representative case among the studied parabens. As can be noted, the hypersurface responses were very similar in all cases, showing a continuous variation with maximum values in a localized spatial area. Figure 2a shows higher EF values when the pH of the donor and acceptor phases increased, with maximum values close to pH 10. At higher pH values could be observed a diminution on the EF, probably due to degradation of the analytes, as corroborated in the HPLC chromatograms. Figure 2d shows the response surfaces for the applied voltage and the extraction time, respectively. Low values for these parameters were associated with poor EF, reaching maximum surface values close to 80 V and 15 min. Higher values no longer produced better EFs, due to the instability of the EME system as well as the appearance of electrolytic degradation products. Figure 2b,c corroborates the findings mentioned above. Similar behavior to that described for PrP was also observed for each of the investigated parabens, both for the response surfaces as well as the involved factors.

According to the experimental design results, pH 10 in donor and acceptor phases, 80 V for the applied voltage, and 15 min extraction time were selected as the best conditions for the EME procedure for the studied compounds. EF values higher than 90 were achieved for all target parabens, except for BuP which showed a lower value.

Finally, analysis of variance (ANOVA) was performed to test the predictive ability of the model. Significant F values were obtained in all cases, with p values less than 0.04. Furthermore, the lack-of-fit test corroborated the appropriate adaptation of the model for each studied compound.

#### 3.2.3. Selectivity Assays

This work aimed at the selective extraction of parabens in the presence of two different drug families (FQs and NSAIDs). Therefore, once optimal conditions for parabens extraction were established, selectivity assays were performed. The EME procedure was then carried out with a donor phase containing three FQs (MARB, ENR and FLM) in addition to the seven worked parabens at the same concentration level (100 µg L^−1^). Under these conditions, the extraction efficiency of parabens remained constant, while the EFs obtained for FQs ranged between 52 and 75. These values indicate that the proposed EME procedure is suitable for the simultaneous extraction of both drug families, which is in accordance with the previous work using a similar EME system for the separately extraction of FQs [10].

In a further step, EME was carried out with a donor phase solution containing the target parabens and FQs, in addition to three NSAIDs (KTP, NAX and SAL, 100 µg L^−1^). EFs for parabens and FQs were as expected, while none of the NSAIDs were extracted. Those results agree with previously reported optimal conditions for the EME of this drug family [19].

Therefore, the selectivity of the method for parabens and FQs versus NSAIDs was assessed, providing the following EFs: 189 for MeP, 168 for EtP, 195 for PrP, 194 for iPrP, 28 for BuP, 94 for iBuP, 111 for BzP, 75 for MRB, 72 for ENR, and 52 for FLM. 

### 3.3. Analytical Performance of the Proposed EME Method

The suitability of the selective EME procedure was studied for the determination of parabens and FQs, fixing the experimental parameters at the optimal conditions described in previous section. As no matrix effect was observed, external calibration was performed. Calibration curves were constructed for each target compound by using least-square linear regression analysis. Linearity, method detection limits (MLODs), method quantitation limits (MLOQs) for each analyte, as well as the repeatability and intermediate precision were evaluated. Figures of merits are summarized in Table 2. 

The linearity percentages, calculated as 100(1 − S_b_/b), where b is the slope of the calibration line of each analyte and S_b_ its standard deviation, were calculated for all the determined compounds [28]. The obtained values for linear ranges were 0.5–500 µg L^−1^ and 1.3–500 µg L^−1^ for the parabens and FQs, respectively. The corresponding values for linearity (%) were higher than 97% for all compounds. Based on a signal-to-noise (S/N) ratio of 3 [29], MLODs were calculated in the ranges 0.2–1.1 µg L^−1^ and 0.4–0.6 µg L^−1^ for parabens and FQs, respectively. For these calculations, the effective recoveries as well as EFs obtained for each analyte were taken into account. In the same way, MLOQs (S/N = 10) ranged between 0.5–3.6 µg L^−1^ for parabens and 1.3–1.9 µg L^−1^ for FQs. Repeatability was calculated, carrying out the EME procedure in triplicate (n = 3) at intermediate precision four times a week, for two weeks (total of 8 extractions). For this purpose, fortified water samples were employed at low, medium, and high concentration levels within the linear range of each analyte. Relative standard deviation (RSD%) values between 4–7% were obtained for repeatability. Intermediate precision data (RSD%) for each of the analyzed compounds were as follows: 8.1% for MeP, 8.3% for EtP, 8.2% for PrP, 8.0% for iPrP, 9.9% for BuP, 8.7% for BzP, 9.6% for MRB, 9.2% for ENR, and 10.0% for FLM, respectively. 

### 3.4. Application to Water Samples

Once the method was validated, the EME procedure was applied to three different types of water samples. For this purpose, samples of river, lake, and swimming pool water were collected in West Andalucía (Spain), described in Section 2.5. After being adjusted to optimum pH 10 (by the addition of aqueous NaOH solution), samples were diluted (1:500) and filtered (through 0.2 μm disposable nylon filters) before the EME procedure was performed, as described in Section 2.2. 

**Figure 3:**
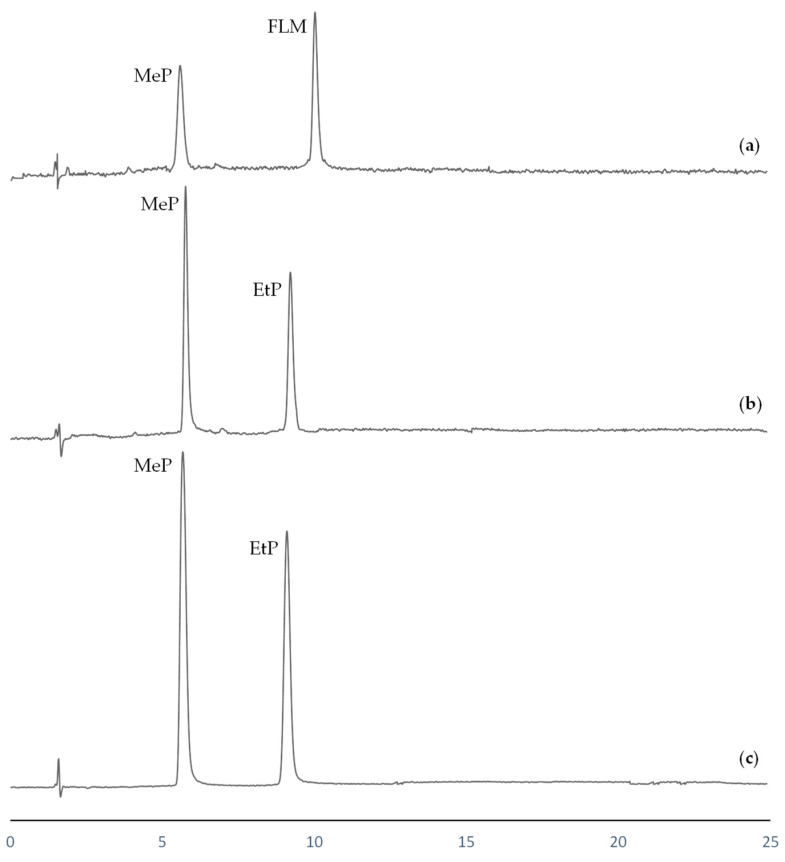
HPLC chromatograms after EME procedure corresponding to (**a**) lake water; (**b**) river water; (**c**) swimming pool water.

Parabens were expected to be present in all analyzed water samples, as they were collected from recreational and aquatic sportive areas frequently used by the public during the summer season. Due to the high temperatures registered in this area, the usage of sunscreens products is common, which are usually prepared with parabens as additives. On analysis, only the most frequently parabens (MeP and EtP) were detected in some of the water samples. Swimming pool water presented concentration levels of 3.2 μgL^−1^ and 1.1 μgL^−1^ of MeP and EtP, respectively. For river water, 1.3 μgL^−1^ of MeP was determined, while the EtP level was under MLOQ. In contrast, in lake water only MeP was detected, and additionally, a high concentration of FLM was also determined in this sample (2.6 μgL^−1^). This fact could be due to the location of this lake in a country area surrounded by several farms, where this drug is often employed as a veterinary antibiotic. HPLC chromatograms corresponding to the analyzed water samples are shown in Figure 3. 

To check the accuracy of the proposed methodology, recovery tests were carried out. Samples were fortified with all the target analytes at three concentration levels (low: 10 µgL^−1^, medium: 100 µgL^−1^, and high level: 250 µgL^−1^), within their respective calibration ranges. Fortified levels were calculated after sample dilution. Recovery values were calculated as follows:

$$R\left(\%\right)=\frac{C_{f}-C_{i}}{C_{a}}100$$

where C_f_ is the final concentration in the acceptor phase extract, C_i_ is the initial concentration in the sample, and C_a_ is the concentration added to the water sample. R values above 81.2% were obtained for all the compounds. These results confirm the suitability of the proposed EME method for the analysis of target analytes in water samples.

### 3.5. Comparative Assesment with Other Miniaturized Procedures 

Regarding the chemical analysis of parabens, only two published works have reported methods involving the use of EME systems [11,30], while most of methods in the literature involve alternative microextraction techniques, such as solid-phase microextraction (SPME), LPME, or dispersive liquid–liquid microextraction (DLLME) procedures. Therefore, the comparative assessment focused on miniaturized protocols for the determination of parabens and FQs [11,30,31,32,33,34], as detailed in Table 3. 

It can be noted that the proposed work provides high preconcentration levels compared with the literature references, enabling an increase in the sensitivity of the method (see MLOD values in Table 2) and the determination of lower amounts of these contaminants in environmental and biological samples.

Furthermore, the chitosan-based EME system has additional important advantages relating to its usage as a support material. In this sense, it enables the simultaneous extraction of a large group of parabens (seven), whereas with the other approaches the number of these compounds is reduced to five, four, or even only three. Meanwhile, the selectivity of this EME system is established, providing the extraction of two different families of compounds (parabens and FQs) in the presence of other drugs (NSAIDs). Conversely, in the previous works the extraction of parabens or FQs was performed as a unique group of analytes, further indicating the versatility of the biopolymeric film as a membrane in this EME procedure. 

The greenness profile of the developed method in comparison to the other reported works is presented in Table 4. As can be noted, the GAPI assessment yields a red-centered pentagram in all cases, reflecting a common negative environmental impact. This fact is mainly due to the characteristics of sample collection, involving an off-line procedure and requiring the transport of the sample to the laboratory. In addition, all methods involve an extraction procedure, which is labelled red in the color code. Among the methods compared, the GAPI pictogram corresponding to the proposed method had the fewest elements in red (only two) and the most in green (up to eight green-filled fields). This greener profile is essentially due to the fact that it is a solvent-free process, which does not require the use of hazardous reagents, with minimal energy consumption and waste generation, and based on the usage of a biodegradable material, i.e., it employs a biopolymeric membrane instead of plastic PP or liquid organic membranes used in other works. As a result, the eco-friendliness assessment using the AGREEprep software also confirms the greener profile of the chitosan-based EME method, with the highest score of 0.7. Approaches enabling in situ sample preparation and supporting automation could even increase this score up to the maximum value of 1 (considered the greenest profile).

## 4. Conclusions

The proposed chitosan-based EME procedure has been successfully demonstrated to enable the selective and simultaneous extraction of two different CECs (parabens and FQs) in the presence of a third family of emerging pollutants (NSAIDs). The greenness of the EME system is an additional feature, as the use of organic-SLM is avoided. Furthermore, suitability of the method for the extraction and determination of target analytes in water samples was assessed, confirming high preconcentration levels and subsequently low MLOQs.

Accordingly, chitosan membrane provides an advantageous material to be used as support in EME systems for the selective determination of CECs in water samples, due to its unique properties: (1) the biopolymeric nature of this material, which confers eco-friendly characteristics compared with commercial plastic-based supports; (2) high versatility, as its chemical composition can be tailored to improve the selective transference of compounds belonging to different families; (3) its active role in the extraction process through targeted interactions biopolymer-analyte, as previously demonstrated; (4) its cationic character, which makes the biomembrane highly conductive, facilitating the extraction procedure.

This important type of biopolymeric support represents a breakthrough in EME development, and its properties will undoubtedly allow its use as a basic platform for designing nanostructured materials in the near future.

## Figures and Tables

**Table 1:** Box–Behnken input matrix employed for the experimental design.

Exp Number	Run Order	Acceptor pH	Donor pH	Voltage (V)	Time (min)
1	7	8	8	85	12.5
2	27	12	8	85	12.5
3	24	8	12	85	12.5
4	10	12	12	85	12.5
5	22	10	10	70	5
6	16	10	10	100	5
7	4	10	10	70	20
8	25	10	10	100	20
9	2	8	10	85	5
10	20	12	10	85	5
11	15	8	10	85	20
12	5	12	10	85	20
13	8	10	8	70	12.5
14	3	10	12	70	12.5
15	6	10	8	100	12.5
16	12	10	12	100	12.5
17	18	8	10	70	12.5
18	26	12	10	70	12.5
19	23	8	10	100	12.5
20	19	12	10	100	12.5
21	11	10	8	85	5
22	21	10	12	85	5
23	1	10	8	85	20
24	13	10	12	85	20
25	14	10	10	85	12.5
26	17	10	10	85	12.5
27	9	10	10	85	12.5

**Table 2:** Figures of merit (linearity, MLOD, MLOQ and EF) for target analytes.

Analyte	Linear Range (µg L^−1^)	Linearity (%)	MLOD (µg L^−1^)	MLOQ (µg L^−1^)	EF
MeP	0.5–500	98.6	0.2	0.5	189
EtP	0.6–500	97.5	0.2	0.6	168
PrP	0.5–500	98.3	0.2	0.5	195
iPrP	0.5–500	98.1	0.2	0.5	194
BuP	3.6–500	97.6	1.1	3.6	28
iBuP	1.1–500	99.1	0.3	1.1	94
BzP	0.9–500	97.8	0.2	0.9	111
MRB	1.3–500	98.5	0.4	1.3	75
ENR	1.4–500	97.3	0.4	1.4	72
FLM	1.9–500	97.0	0.6	1.9	52

**Table 3:** Comparison of the proposed chitosan-based EME method with other reported microextraction procedures for the determination of parabens and FQs. HPLC-DAD determination was performed in all the compared methods.

Target Analytes	Solid Support/Membrane	Solvent/SLM	Extraction Method and Conditions	Extraction Efficiency		MLOD (µgL^−1^)	MLOQ (µgL^−1^)	Matrix	Ref.
				EF	R (%)				
MeP, EtP, PrP	Stainless steel mesh	DES	TF-SPME Sample: pH 5 (0.06 g mL^−1^ NaCl) 40min, 120 rpm (adsorption), 10min, 200 rpm (desorption)	166–183	−	0.018–0.055	0.06–0.182	Lake and river waters	[31]
MeP, EtP, PrP, BuP	None	MIL (extraction), ACN (dispersion)	SA-DLLME 1min, vortex	−	82.0–114.6	0.6–0.84	2.0–2.8	Tap, lake, and river waters	[32]
EtP, PrP, BuP, iBuP, BzP	PP Accurel^®^ S6/2 hollow fiber	1-octanol	HF-EME DP: pH 4 AP: pH 12 30V, 40min, 300 rpm	30–49	−	0.98–1.43	−	Surface environmental water	[11]
EtP, BuP, iBuP	PP flat membrane	1-octanol	Microfluidic-EME DP: pH 3 AP: pH 11.5 4V, 10min-SLM, 7min-dynamic mode	−	100.6–104.2	70–120	240–380	Urine	[30]
MRB, NRF, CPR, DNF, ENR, GTF, GRP	PP Accurel^®^ S6/2 hollow fiber	1-octanol	HF-EME DP: pH 5 AP: pH 2 50V, 15min, 750 rpm	40–85	−	0.005–0.07	0.007–0.15	Urban wastewaters	[33]
ENO, LEV, NRF, CPR, ENR	None	MIL (extraction)	VA-DLLME	19–25	−	0.75–1.5	2.5–5.0	Tap water, milk, honey, chicken	[34]
MeP, EtP, PrP, iPrP, BuP, iBuP, BzP, MRB, ENR, FLM	Chitosan flat membrane	SLM-free	EME DP: pH 10 AP: pH 10 80V, 15min, 600 rpm	28–195	−	0.2–1.1	0.5–3.6	Environmental water (in the presence of NSAIDs)	This work

Definitions: TF-SPME: thin-film solid-phase microextraction; MIL: magnetic ionic liquid; SA-DLLME: salting-out assisted dispersive liquid–liquid microextraction; NRF: norfloxacin; CPR: ciprofloxacin; DNF: danofloxacin; GTF: gatifloxacin; GRP: grepafloxacin; ENO: enoxacin; LEV: levofloxacin; VA-DLLME: vortex-assisted dispersive liquid–liquid microextraction.

**Table 4:** Greenness assessment of the proposed chitosan-based procedure and the other systems compared.

Miniaturized Method	GAPI Assessment	AGREEprep Assessment	Ref.
TF-SPME	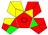	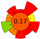	[31]
SA-DLLME	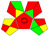	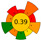	[32]
HF-EME	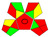	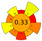	[11]
Microfluidic-EME	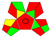	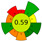	[30]
HF-EME	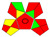	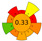	[33]
VA-DLLME	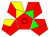	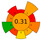	[34]
Chitosan-EME	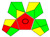	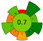	This work

## Data Availability

Not applicable.

## Supplementary Materials

The following supporting information can be downloaded at: https://www.mdpi.com/article/10.3390/membranes13030326/s1, Figure S1: EME device; Table S1: Gradient conditions for the chromatographic analysis. A: 28 mM aqueous phosphate buffer pH 2.5; B: Methanol; Table S2: Chemical structures and pKa values of target analytes.

## References

[1] Barceló D. (2003). Emerging pollutants in Water Analysis. TrAC Trends Anal. Chem..

[2] White paper aquatic life criteria for contaminants of emerging concern—Part I—General Challenges and Recommendations. Prepared by the OW/ORD Emerging Contaminants Workgroup 3 June 2008. https://www.epa.gov/wqc/contaminants-emerging-concern-including-pharmaceuticals-and-personal-care-products.

[3] Morales-Paredes C., Rodríguez-Díaz J., Boluda-Botella N. (2022). Pharmaceutical compounds used in the COVID-19 pandemic: A review of their presence in water and treatment techniques for their elimination. Sci. Total Environ..

[4] Li J., Jiang Y., Sun Y., Wang X., Ma P., Song D., Fei Q. (2022). Extraction of parabens by melamine sponge with determination by high-performance liquid chromatography. J. Sep. Sci..

[5] Shishov A., Gerasimov A., Nechaeva D., Volodina N., Bessonova E., Bulatov A. (2020). An effervescence-assisted dispersive liquid–liquid microextraction based on deep eutectic solvent decomposition: Determination of ketoprofen and diclofenac in liver. Microchem. J..

[6] Chen J., Deng W., Li X., Wang X., Xiao Y. (2019). Hexafluoroisopropanol/Brij-35 based supramolecular solvent for liquid-phase microextraction of parabens in different matrix samples. J. Chromatogr. A.

[7] Barahona F., Albero B., Tadeo J., Martín-Esteban A. (2019). Molecularly imprinted polymer-hollow fiber microextraction of hydrophilic fluoroquinolone antibiotics in environmental waters and urine samples. J. Chromatogr. A.

[8] Drouin N., Kubáň P., Rudaz S., Pedersen-Bjergaard S., Schappler J. (2019). Electromembrane extraction: Overview of the last decade. TrAC Trends Anal. Chem..

[9] Aranda-Merino N., Ramos-Payán M., Callejón-Mochón M., Villar-Navarro M., Fernández-Torres R. (2020). Comparison of three electromembrane-based extraction systems for NSAIDs analysis in human urine samples. Anal. Bioanal. Chem..

[10] Román-Hidalgo C., Aranda-Merino N., López-Pérez G., Sánchez-Coronilla A., Villar-Navarro M., Martín-Valero M. (2021). Chitosan biofilms: Insights for the selective electromembrane extraction of fluoroquinolones from biological samples. Anal. Chim. Acta.

[11] Villar-Navarro M., Moreno-Carballo M.C., Fernández-Torres R., Callejón-Mochón M., Bello-López M. (2016). Electromembrane extraction for the determination of parabens in water simples. Anal. Bioanal. Chem..

[12] Li J., Zhu R., Shen X., Huang C. (2022). Functional materials and chemicals in electromembrane extraction. TrAC Trends Anal. Chem..

[13] Santos L.B., Assis R.S., Barreto J.A., Bezerra M.A., Novaes C.G., Lemos V.A. (2022). Deep eutectic solvents in liquid-phase microextraction: Contribution to green chemistry. TrAC Trends Anal. Chem..

[14] Eie L.V., Pedersen-Bjergaard S., Hansen F.A. (2022). Electromembrane extraction of polar substances—Status and perspectives. J. Pharm. Biomed. Anal..

[15] Sedehi S., Tabani H., Nojavan S. (2018). Electro-driven extraction of polar compounds using agarose gel as a new membrane: Determination of amino acids in fruit juice and human plasma samples. Talanta.

[16] Román-Hidalgo C., Ramos-Payán M., Ocaña-González J., Martín-Valero M., Bello-López M. (2015). Agar films containing silver nanoparticles as new support for electromembrane extraction. Anal. Bioanal. Chem..

[17] Asadi S., Tabani H., Nojavan S. (2018). Application of polyacrylamide gel as a new membrane in electromembrane extraction for the quantification of basic drugs in breast milk and wastewater samples. J. Pharm. Biomed. Anal..

[18] Hajizadeh S., Farhadi K., Molaei R., Forough M. (2020). Silver nanoparticles-tragacanth gel as a green membrane for effective extraction and determination of capecitabine. J. Separ. Sci..

[19] Román-Hidalgo C., López-Pérez G., Martín-Valero M.J., Bello-López M. (2019). Chitosan tailor-made membranes as biopolymeric support for electromembrane extraction. Talanta.

[20] Gholami H., Ghaedi M., Arabi M., Ostovan A., Bagheri A., Mohamedian H. (2019). Application of Molecularly Imprinted Biomembrane for Advancement of Matrix Solid-Phase Dispersion for Clean Enrichment of Parabens from Powder Sunscreen Samples: Optimization of Chromatographic Conditions and Green Approach. ACS Omega.

[21] Román-Hidalgo C., López-Pérez G., Villar-Navarro M., Martín-Valero M. (2023). Green electromembrane extraction procedure based on biodegradable chitosan films for determination of polyphenolic compounds in food samples: Greenness assessment of the sample preparation approach. Talanta.

[22] Płotka-Wasylka J. (2018). A new tool for the evaluation of the analytical procedure: Green Analytical Procedure Index. Talanta.

[23] Wojnowski W., Tobiszewski M., Pena-Pereira F., Psillakis E. (2022). AGREEprep—Analytical greenness metric for sample preparation. TrAC Trends Anal. Chem..

[24] Katakam L.N.R., Ettaboina S.K., Dongala T. (2021). A simple and rapid HPLC method for determination of parabens and their degradation products in pharmaceutical dosage forms. Biomed. Chromatogr..

[25] Tartaglia A., Kabir A., Ulusoy S., Sperandio E., Piccolantonio S., Ulusoy H.I., Furton K.G., Locatelli M. (2019). FPSE-HPLC-PDA analysis of seven paraben residues in human whole blood, plasma, and urine. J. Chromatogr. B.

[26] Yamini Y., Seidi S., Rezazadeh M. (2014). Electrical field-induced extraction and separation techniques: Promising trends in analytical chemistry—A review. Anal. Chim. Acta.

[27] Tabani H., Fakhari A.R., Shahsavani A. (2013). Simultaneous determination of acidic and basic drugs using dual hollow fibre electromembrane extraction combined with CE. Electrophoresis.

[28] Cuadros L., García A., Bosque J. (1996). Statistical estimation of linear calibration range. Anal. Lett..

[29] Miller J., Miller J. (2000). Statistics and Chemometrics for Analytical Chemistry.

[30] Santigosa-Murillo E., Muñoz-Berbel X., Maspoch S., Muñoz M., Ramos-Payán M. (2020). Impedance model for voltage optimization of parabens extraction in an electromembrane millifluidic device. J. Chromatogr. A.

[31] Werner J., Zgoła-Grześkowiak A., Grześkowiak T. (2022). Development of novel thin-film solid-phase microextraction materials based on deep eutectic solvents for preconcentration of trace amounts of parabens in surface waters. J. Sep. Sci..

[32] Tao Y., Jia L., Qin H., Niu R., Qiao L. (2022). A new magnetic ionic liquid based salting-out assisted dispersive liquid–liquid microextraction for the determination of parabens in environmental water samples. Anal. Methods.

[33] Ramos-Payán M., Villar-Navarro M., Fernández-Torres R., Callejón-Mochón M., Bello-López M.Á. (2013). Electromembrane extraction (EME)—An easy, novel and rapid extraction procedure for the HPLC determination of fluoroquinolones in wastewater samples. Anal. Bioanal. Chem..

[34] Qiao L., Tao Y., Yao W., Zhao J., Yan Y. (2022). A magnetic ionic liquid based vortex-assisted dispersive liquid-liquid microextraction coupled with back-extraction for the enrichment of fluoroquinolone antibiotics. J. Pharm. Biomed. Anal..

