# Supplementary material for: Green Method for the Selective Electromembrane Extraction of Parabens and Fluoroquinolones in the Presence of NSAIDs by Using Biopolymeric Chitosan Films
Source: Membranes (Basel). 2023 Mar 12;13(3):326. doi: 10.3390/membranes13030326 (PMC10059583; doi:10.3390/membranes13030326)
Supplement: Supplementary file 1 [file membranes-13-00326-s001.zip › membranes-2240857-supplementary.pdf]

Supplementary Material

Green Method for the Selective Electromembrane Extraction of Parabens and Fluoroquinolones in the Presence of NSAIDs by Using Biopolymeric Chitosan Films

Cristina Román-Hidalgo [1], María Jesús Martín-Valero [1,*], Germán López-Pérez [2] and Mercedes Villar-Navarro [1,*]

[1] Department of Analytical Chemistry, Faculty of Chemistry, Universidad de Sevilla, c/Prof. García González, s/n. 41012-Seville, Spain;

[2] Department of Physical Chemistry, Faculty of Chemistry, Universidad de Sevilla, c/Prof. García González, s/n. 41012-Seville, Spain;

* Correspondence: mmartin@us.es (M.J.M.-V.); mvn@us.es (M.V.-N.); Tel.: +34-954556308 (M.J.M.-V.); +34-954557172 (M.V.-N.)

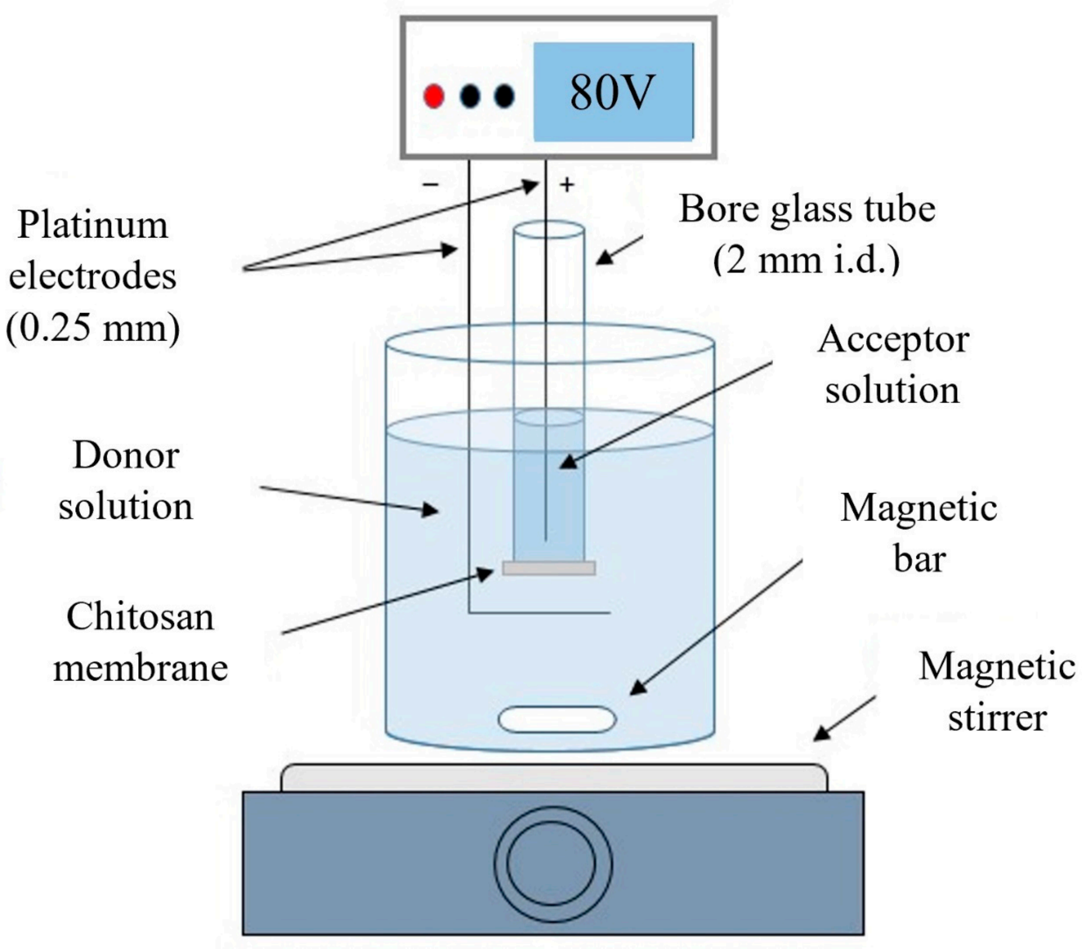

Figure S1. EME device.

Table S1. Gradient conditions for the chromatographic analysis. A: 28mM aqueous phosphate buffer pH 2.5; B: Methanol.

t (min)	%A	%B
0	55	45
2	50	50
5	50	50
6	45	55
10	45	55
14	40	60
22	40	60
23	15	85
25	15	85

Table S2. Chemical structures and pKa values of target analytes.

Analyte	Chemical Structure	pKa
MeP	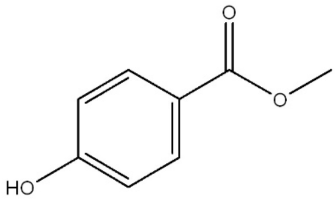	8.17
EtP	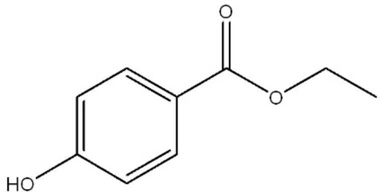	8.22
iPrP	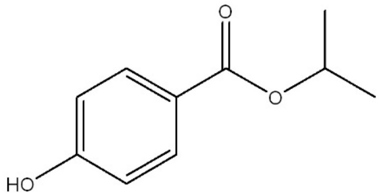	8.4
PrP	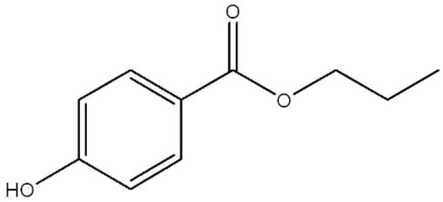	8.35
BzP	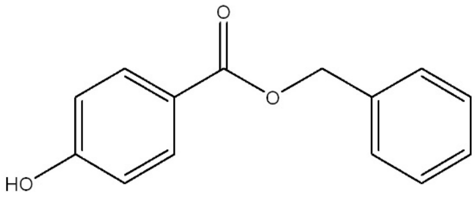	8.18
iBuP	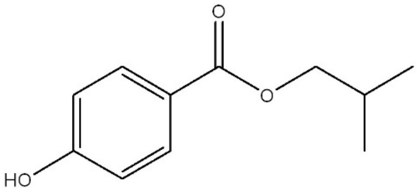	8.17

BuP	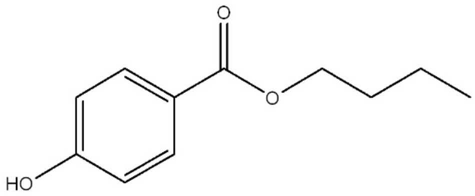	8.37
MBR	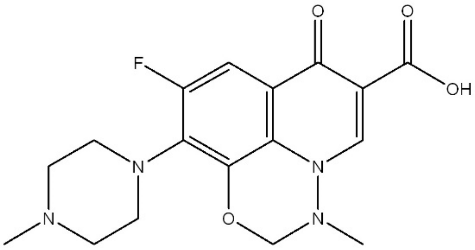	5.7; 8.9
ENR	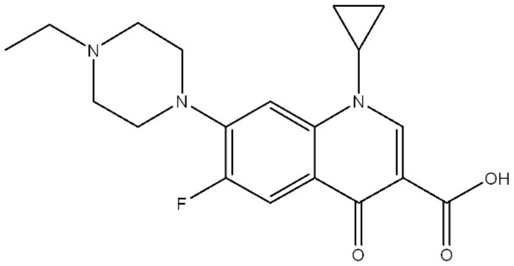	7.9; 6.2
FLM	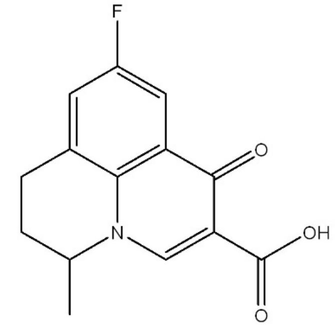	6.5
SAL	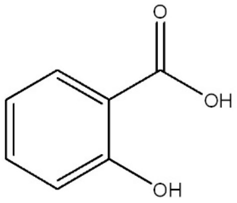	2.3; 13.4
KTP	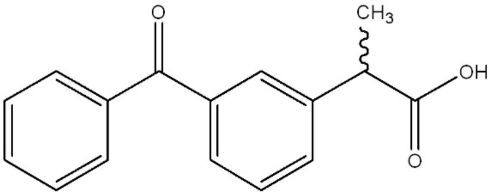	4.23
NAX	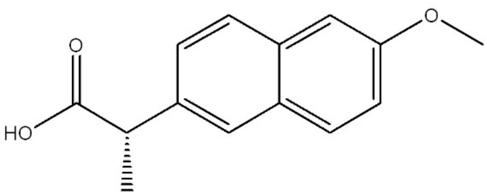	4.84
